# Spectral Skyline Separation: Extended Landmark Databases and Panoramic Imaging

**DOI:** 10.3390/s16101614

**Published:** 2016-09-29

**Authors:** Dario Differt, Ralf Möller

**Affiliations:** Computer Engineering Group, Faculty of Technology, Bielefeld University, D-33594 Bielefeld, Germany; moeller@ti.uni-bielefeld.de

**Keywords:** UV, color vision, insect vision, linear separation

## Abstract

Evidence from behavioral experiments suggests that insects use the skyline as a cue for visual navigation. However, changes of lighting conditions, over hours, days or possibly seasons, significantly affect the appearance of the sky and ground objects. One possible solution to this problem is to extract the “skyline” by an illumination-invariant classification of the environment into two classes, ground objects and sky. In a previous study (Insect models of illumination-invariant skyline extraction from UV (ultraviolet) and green channels), we examined the idea of using two different color channels available for many insects (UV and green) to perform this segmentation. We found out that for suburban scenes in temperate zones, where the skyline is dominated by trees and artificial objects like houses, a “local” UV segmentation with adaptive thresholds applied to individual images leads to the most reliable classification. Furthermore, a “global” segmentation with fixed thresholds (trained on an image dataset recorded over several days) using UV-only information is only slightly worse compared to using both the UV and green channel. In this study, we address three issues: First, to enhance the limited range of environments covered by the dataset collected in the previous study, we gathered additional data samples of skylines consisting of minerals (stones, sand, earth) as ground objects. We could show that also for mineral-rich environments, UV-only segmentation achieves a quality comparable to multi-spectral (UV and green) segmentation. Second, we collected a wide variety of ground objects to examine their spectral characteristics under different lighting conditions. On the one hand, we found that the special case of diffusely-illuminated minerals increases the difficulty to reliably separate ground objects from the sky. On the other hand, the spectral characteristics of this collection of ground objects covers well with the data collected in the skyline databases, increasing, due to the increased variety of ground objects, the validity of our findings for novel environments. Third, we collected omnidirectional images, as often used for visual navigation tasks, of skylines using an UV-reflective hyperbolic mirror. We could show that “local” separation techniques can be adapted to the use of panoramic images by splitting the image into segments and finding individual thresholds for each segment. Contrarily, this is not possible for ‘global’ separation techniques.

## 1. Introduction

### 1.1. Motivation

The remarkable ability of social insects to navigate between important locations, e.g., the nest and a feeding site, has been the subject of many studies [1,2,3,4]. Several navigational strategies have been unraveled experimentally, which explain the observed navigation abilities of insects, including pheromone trails, path integration and visual landmark navigation. Especially the latter not only receives attention in biology, but also in many technical fields like autonomous driving, localization and route following of driver-less cars and robots [5,6]. In this work, we focus on extracting the skyline from camera images as a basis for visual navigation tasks.

Desert ants have been studied extensively as animal models for visual navigation strategies [10,11]. Due to the high ground temperatures, pheromone trails are not persistent in desert environments, such that these insects have to rely on path integration and visual information. For visual input, it is assumed that these insects use the polarization pattern of the sky as a compass for path integration and salient landmarks and the skyline as input for visual landmark navigation (reviews: [1,12,13]). However, the use of salient landmarks suffers from being dependent on the changing appearance of the visual scene: changes of the weather conditions or time of day make the comparison between remembered and currently-perceived landmarks difficult. The binary skyline, comprising uniformly-dark objects in front of a uniformly-bright sky, is a holistic visual scene description that, assuming it can be extracted from the visual input of an insect, has the advantage that it is independent of illumination conditions caused by varying weather and time. However, due to the limited computational power of insect brains, the extraction has to be based on a simple mechanism to be considered as an explanation of insect landmark navigation abilities.

**Figure 1:**
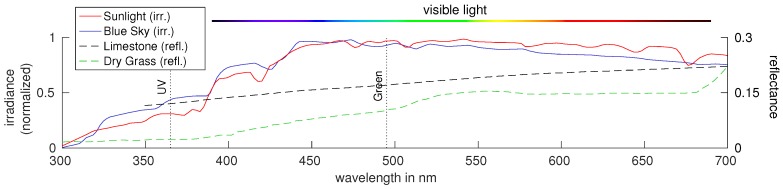
Irradiance spectrum (solid lines) of sunlight [7] (red), clear blue sky [8] (blue) and reflectance spectra (dashed lines) of limestone [9] (black) and dry grass [9] (green). The peaks of the glass filters used to obtain images in the UV and green channel are depicted (dotted lines). Note that the green channel is inspired by the insect *Cataglyphis bicolor* and does not match exactly with the perception of colors by humans (color bar). [Figure created by author from data in [7,8,9].]

For desert ants of the species *Cataglyphis bicolor*, it is known that light in two different color channels in the UV (≈350 nm) and green (≈500 nm) range (Figure 4) is perceived [14]. While ants with trichromatic color vision have been found (*Myrmecia vindex* [15]), which have an additional blue color channel (≈450 nm), they share the UV and green color channel. Figure 1 shows irradiance spectra of direct sunlight and blue sky, as well as reflectance spectra of limestone and dry grass. For objects on the ground, the reflectivity in the UV channel is commonly small (especially compared to visual light), while the UV portion of direct sun light and blue sky is high. Therefore, there is a high contrast between sunlight reflected from ground objects and the sky. As proposed by Wehner [16], this allows a simple classification between ground objects and the sky (resulting in a binary skyline) in the UV channel by determining an appropriate threshold. This idea is supported by behavioral experiments with the ant *Melophorus bagoti*, where it could be shown that their navigational abilities are significantly decreased as soon as UV light is blocked [17]. The lower reflectivity of ground objects in the UV channel compared to the green channel motivates an alternative approach: instead of only using the UV channel for skyline extraction, a contrast of the UV and green channel can be used [11]. The spectral characteristics of ground objects under natural illumination, as well as the difference in the spectra of light emitted directly from the sun and diffuse lighting from the sky have been examined by [11,18]: using a handheld device containing two photodiodes sensitive to UV and green light, databases have been collected, which contain log UV/G data for a wide range of ground objects and sky patches. The data show that a simple linear separation of the logarithmized UV/G data (in the following denoted as log UV/G) is sufficient to correctly classify most of the collected data into two classes, ground objects and sky. Since the databases contain objects and sky patches recorded under different lighting and weather conditions, this suggests that, using an appropriate log UV/G contrast mechanism, a threshold can be found to reliably extract the skyline.

**Figure 2:**
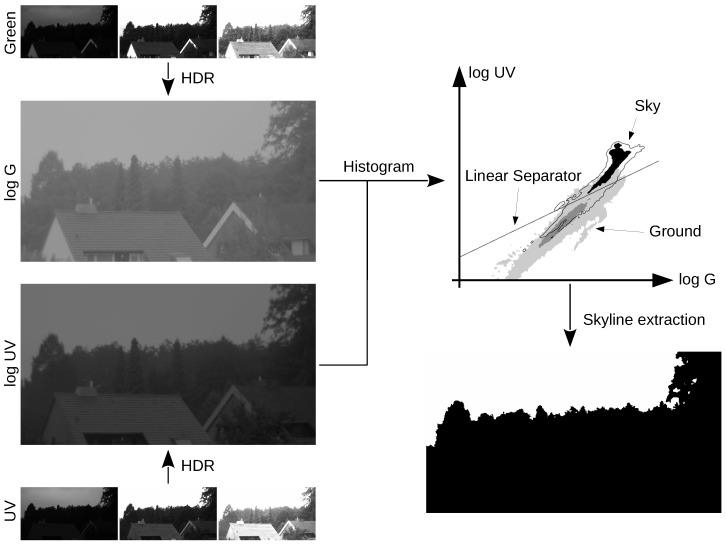
Overview of the different processing steps: For each channel (UV and green) the raw input images with varying exposure times (left, small images) are combined into HDR images (left, large images). Afterwards, a 2D histogram is built (right, top), showing the distribution of the log UV and log G values for each pixel of either a database of multiple image pairs (“global” methods) or of a single image pair (“local” methods). Finally, a linear separator is used to classify each pixel as either ground or sky, providing an image of the skyline (right, bottom) which is widely illumination invariant.

**Figure 3:**
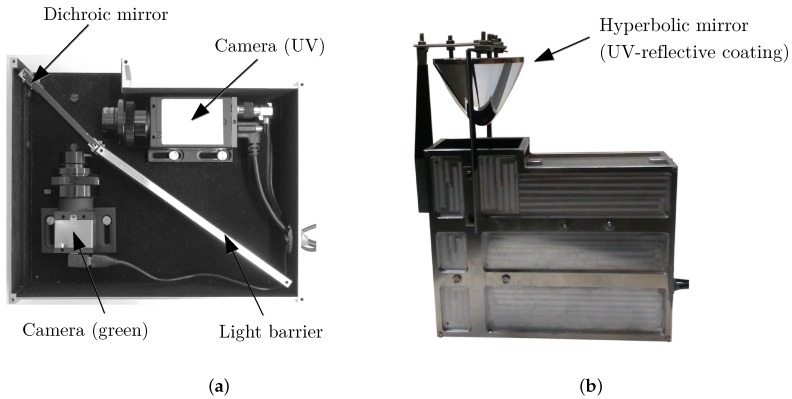
(**a**) The experimental setup (cover-plate removed) with two cameras recording the UV- and green-channel simultaneously; from [19]. (**b**) To capture panoramic images, a hyperbolic mirror can be mounted. The mirror has a reflective (aluminum) and a protective layer (magnesium fluoride) to provide a nearly constant reflection over a wide range of wavelengths (including UV and visible light).

In comparison to the former studies using sensors with a single photodiode per channel, in this study (and our previous study [19]), we use a camera setup that allows the acquisition of complete log UV/G images instead of single log UV/G data points. The photodiode sensors had an opening angle of around 10 degrees, such that precise measurements of single objects were not possible, contrary to the camera setup that allows one to collect precise measurements of objects and is only limited by the resolution of the cameras used. Figure 2 shows a flow chart of the processing steps leading from the raw images captured by our camera setup (Figure 3) to a binary classification of the scene. Moreover, the camera setup was used to record irradiance values in both the UV and green channel over complete days, while the data collected by the photodiode sensors covered a more restricted time range.

### 1.2. Global and Local Classification Methods

All methods tested in this study are based on the same idea (Figure 2 and Figure 8): First, the HDR (high dynamic range) image data (UV and green) of all pixels are projected onto a one-dimensional plane (contrast mechanism). For all tested methods in this study, we assume that the projection plane is fixed and needs to be learned. Note that in our previous study [19] we also examined local separation techniques (unsupervised) that learned both the projection angle and the threshold value. Due to their comparable weak performance (also on the newly-collected databases), they are not considered in this study (Section 2.3). Second, we determine a threshold value to perform the classification between ground objects and the sky. There are mainly two approaches for the second step:

- Local: Unsupervised methods, where the threshold is learned for each HDR image pair (UV and green) individually.
- Global: Supervised methods, where the threshold is learned offline on a given, hand-labeled dataset.

For local methods, a classification of ground objects and the sky can be obtained by calculating the histogram of the projected data and determining an appropriate threshold value. While the implementation of thresholding algorithms is simple, the determination of the threshold value may be a rather complex task for insects. Whether biologically-plausible versions of local methods can be found is currently open.

Global methods use a fixed threshold value that is learned on a large set of training data. For global methods, the classification between ground objects and the sky reduces to the calculation of a scalar product and a simple comparison (Section 2.3). Such a separation would be computationally inexpensive and could be accomplished by a simple neuronal network, making it a plausible model for skyline extraction in insects. Both local and global methods use a fixed projection angle that is learned (supervised) from hand-labeled data in our study. In a neural model, the projection (scalar product) could be encoded by fixed synaptic weights.

### 1.3. Related Work

Local methods have been successfully used to extract the skyline using only the UV channel only on mobile robots [6,20]. However, in these studies, skyline extraction was mainly performed in suburban environments or in forests. While these environments were covered in our previous study [19], this study aims to examine the possible application of skyline extraction also in mineral-rich environments, like deserts, rocky landscapes or beaches.

As an alternative to the UV channel, it has been suggested to use the infrared channel [21] or visual light [22] for skyline extraction. These methods optimize energy functions to find an optimal skyline, a rather complex approach that is computationally expensive (compared to simple thresholding) and not feasible as a model for insects.

Besides skyline extraction, several applications have been found for using UV imagery in outdoor robotic experiments: Based on the observation that many insects use UV and green receptors to detect polarization, the detection of polarization patterns in sky has been tested in both channels [23]. They also used that in UV images clouds are nearly invisible and that the contrast between ground objects and sky is increased to improve the distinction between ground objects and the sky. This observation is, for example, also used to estimate the attitude [24] or to correct the drift of the gyroscopes [25] of unmanned aerial vehicles (UAV).

### 1.4. Contributions

In a previous study [19], we could show that local and global methods can be used to perform skyline extraction in a suburban/forest environment. Especially, we could show that by using only the UV channel, the quality of skyline extraction was only slightly worse compared to using information from both the UV and green channel. However, the collected data were limited to a specific environment, and the camera used could not collect omnidirectional images. In this study, we address these limitations by conducting three additional experiments.

First, we collected three additional datasets of mineral landmarks. For each dataset, we recorded the appearance of a skyline made of stones, sand and earth (each over a complete day) in the UV and green channel. Combined with the forest/suburban database from our previous study, we increase the amount of collected sky data to a total of ten days, including dry and sunny days, as well as rainy days. The mineral skyline databases greatly increase the variety of spectral characteristics for objects an insect or robot might encounter in outdoor environments. On these datasets we repeat the experiments from our previous study and examine if skyline extraction can be performed using local and global separation techniques.

Second, we collected a wide variety of ground objects (e.g., undergrowth, shrubs, pavement, etc.) to further increase the database of potential landmarks. Some of these ground objects were collected under three different lighting conditions (directly exposed to the sun, in the shadow during a sunny day, diffusely lighted during a cloudy day) to show the influence of lighting conditions on ground objects. We show that especially diffusely-illuminated minerals, e.g., stones lying in shadows, increase the difficulty to classify ground objects and sky. Moreover, we show that the spectral characteristics of most objects that could be encountered by insects or robots do not differ noticeably from the data collected in the skyline datasets, extending the validity of our results to a wider range of environments.

Third, omnidirectional images have been collected to show the influence of the varying spectral irradiance over the sky. As we will see, for global and local methods, the classification quality is strongly decreased for omnidirectional images. While local methods can easily be adapted by splitting the image into multiple segments and using an individual threshold for each, the adaption of global methods is not possible without violating the idea of a simple classification as it could be used by insects. Calculating thresholds for multiple segments also reduces the effect of lens flare to the process of skyline extraction, which may lead to errors during the process of skyline extraction [6]. Moreover, for omnidirectional images (e.g., using a fish eye lens), the skyline can be extracted completely and allows the use of existing navigational algorithms for panoramic images [26,27,28].

Summarized, this study aims to answer two questions: on the one hand, we want to answer the question if insects could benefit from multi-spectral vision as suggested by [11] (here: UV/G) to extract the skyline as an illumination-invariant landmark using parsimonious algorithms; and on the other hand, if local methods yield robust and computationally-inexpensive (omnidirectional) skyline extraction usable for robot navigation.

## 2. Materials and Methods

In this section, we will summarize the process of image acquisition, as well as the visualization and classification of these data.

### 2.1. Image Acquisition

**Figure 4:**
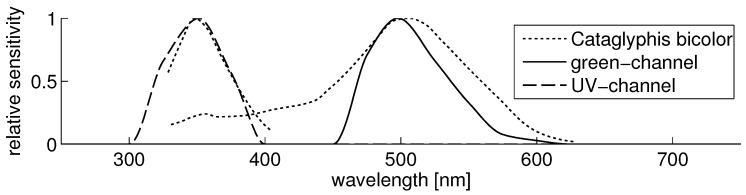
The plot shows the normalized relative sensitivities resulting from combining all optical components. For comparison, the spectral sensitivity of UV and green photoreceptors of the desert ant *Cataglyphis bicolor* [14] are plotted as dotted lines. [Figure created by author from data in [14].]

We built a setup containing two cameras, sensitive to UV and visible light, respectively, which are separated by a dichromatic mirror (Figure 3), allowing both cameras to record the same scene simultaneously. The cameras have been equipped with additional filters to limit the received range of light, that is 350±50 nm for the UV camera and 500±70 nm (green) for the other camera. The resulting relative sensitivities with all optical components involved (cameras, filters, mirror) can be seen in Figure 4. For detailed information (e.g., individual spectra for the used filters, mirrors and cameras), see [19].

Both cameras take multiple images with varying exposure times ($2^{k}$ ms with k=−3⋯7) of each scene. These images are then fused to a high dynamic range (HDR) image containing the logarithmic irradiance information for each pixel of the scene using a slightly modified form of the algorithm described by [29]; see [19]. The logarithmic scaling is inspired by the response of photoreceptors in the eye to increasing irradiance values as observed for insects [30,31] or humans [32,33]. Alternatively to a logarithmic scaling, the linear camera response could be used to increase the color contrast in images [34,35]. However, a logarithmic representation has two advantages over linear camera response: First, a large range of irradiance values (here: 14 camera stops) can be represented visually. Second, a log UV/G contrast, robust to global intensity changes [11], can be implemented as a linear separator (Section 2.3).

The resulting images cover a wide range of light intensities, such that all collected data (from sunrise to sunset and over several days) share the same range of irradiance values.

**Figure 5:**
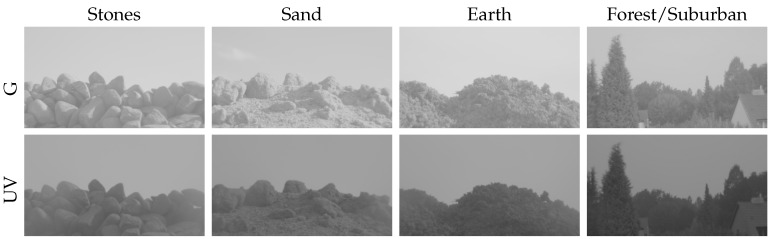
Examples of captured HDR images with our camera setup taken at 17:00 for both the UV and green channel. The first three columns show different mineral skylines (stones, sand, earth), while the last column shows a skyline in a suburban area of Bielefeld (forest/suburban).

In the previous study, we collected data over seven subsequent days from 31 August 2014 to 6 September 2014 in a suburban area of Bielefeld (Germany), where a set of UV and green HDR images has been recorded in 5-min intervals over each complete day. These data contained mainly natural objects, like trees and shrubs, as well as some artificial objects, like house walls and roofs. Over the seven days, a wide variety of different weather conditions could be observed: three days were sunny with sunshine durations between 5 and 11 h, while another three days were dominated by cloudy skies (0–3.5 h sunshine duration) with a little rain. The last day was recorded on a sunny day, on which a strong summer storm with excessive rain occurred in early afternoon (skyline databases suburban/forest; Figure 5).

**Figure 6:**
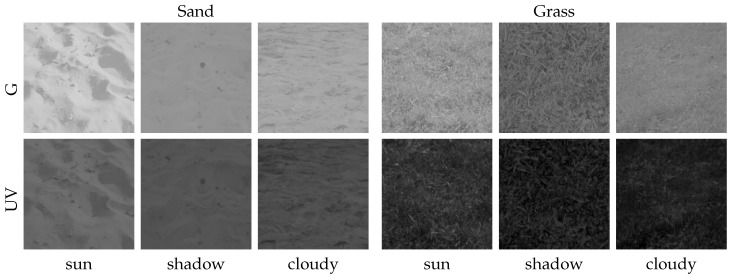
Single HDR images of sand and grass captured with our camera setup for both the UV and green channel. A total of three different images have been taken for each object: Two images were taken on a sunny day, one time where the object is exposed to the sun (sun) and one time lying in the shadows (shadow). A third one was collected during a cloudy day (cloudy).

**Figure 7:**
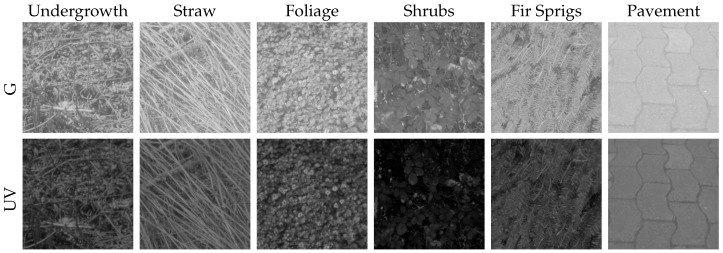
Examples of further collected data in the object database, which were not explicitly collected under specific lighting conditions as the data presented in Figure 6.

In this study, we extend the variety of ground objects by including two additional datasets: first, we collected another set of data over three days between 23 August 2015 and 29 August 2015. We created three different skylines in front of the camera, formed by (dry) stones, sand and earth. We were not able to add a cover for our non-waterproof camera setup, as this would have influenced the lighting conditions of the skyline. We therefore could not record images on days with rain or high humidity. The sunshine duration on the recording days differed between 9 and 12 h (skyline databases sand/earth/stone; Figure 5). To avoid direct incident sunlight on the camera, which could damage the sensors or lead to over-exposed images, the camera setup was pointing north in all recordings of the skyline databases. Second, we collected a total of 61 records with a wide variety of ground objects (without sky). Some of these objects (patches of grass, trees, stones, gravel, earth and sand) have been recorded under three different lighting conditions: objects (a) lying in the shadow on a sunny day; (b) directly exposed to sunlight on a sunny day and (c) exposed to diffuse sky light on a cloudy day. The remaining objects recorded (without specific lighting conditions) contain foliage, fir sprigs, shrubs, pavement, undergrowth and straw to ensure a wide range of collected data (object database; Figure 6 and Figure 7).

Additionally, we collected a total of 12 panoramic images on 12 May 2016 and 27 June 2016 during sunny weather to examine the influence of direction-dependent lighting. The panoramic images were recorded using a self-crafted hyperbolic mirror mounted in front of the experimental setup (Figure 3). The hyperbolic mirror has a reflective (aluminum) and a protective layer (magnesium fluoride) to provide a nearly constant reflection over a wide range of wavelengths (including UV and visible light). The mirror has a maximal opening angle of around $-20^{\circ }$–$45^{\circ }$ relative to the horizon. For each panoramic image, we manually created a mask to mask out areas from the panoramic images that are corrupted by direct incident sunlight on the camera.

### 2.2. Data Visualization

The recorded images contain the logarithmic UV and green irradiance values for each single pixel after the HDR algorithm has been applied; also in animals, the perception of irradiance values generally follows a logarithmic scaling [30,31]. We will in the following refer to these data pairs as log UV and log G data (or log UV/G data). By normalizing the log UV/G data to the minimal and maximal observed values (individually for each channel), we can map the log UV/G data onto the interval [0,255]⊆N, allowing the usage of discrete methods and visualization as images.

By super-positioning a narrow Gaussian distribution (σ=1) for an arbitrary set *X* of log UV/G data into a plot with the log G values on the *X*-axis and log UV values on the *Y*-axis, a continuous distribution of the log UV/G data can be visualized (in the following called log UV/G plots). Since the classification as a ground object or sky is available for all recorded log UV/G data (Section 2.3), we can plot a distribution for both classes. For better comparison, the log UV/G plots are normalized to the interval [0,1], where 1 is the maximum value of both distributions. In all of the plots, the levels 0.01 and 0.1 are highlighted (Figure 9 and Figure 10) to get a better visual impression of the data.

We will denote by $X_{t_{1}-t_{2}}$ the log UV/G data recorded between the specified hours $t_{1}$ and $t_{2}$. Therefore, we will denote by $X_{8–19}$ and $X_{7–20}$ the log UV/G data recorded between 8:00–19:00 and 7:00–20:00, respectively. We chose these two specific day times for two reasons: First, all separation techniques show a stable performance between 8:00 and 19:00 due to the moderate changes of the lighting conditions, in contrast to the strong changes between 7:00–8:00 and 19:00–20:00 (beginning sunset/sunrise in our datasets). Second, the classification rates of all tested global separation methods drop noticeably outside of these day times [19]. For visualization, as well as data classification, we will use samples *X* that have a total of $10^{5}$ log UV/G data points of both classes ground objects and sky.

### 2.3. Data Classification

Since we are interested in examining different methods for classifying the recorded log UV/G data into ground objects and sky, we classify the recorded data manually to get a ground truth for further evaluation. For a set *X* of log UV/G data, we denote by:

(1) $$R=\frac{\left\{x\in X\;|\;x\;\mathrm{correctly}\;\mathrm{classified}\right\}}{\left|X\right|}$$

the classification rate, where |X| denotes the number of elements in *X*. All methods tested in this study to classify the log UV/G data as ground object or sky, in the following called separation techniques, are evaluated using the classification rate as a measure. As in the previous study [19], we will examine the performance of two different types of separation techniques, called global and local (Section 1.2).

A linear separator is, in the case of the log UV/G data, a 2-dimensional vector $s\in R^{2}$ with ∥s∥=1 (equivalently represented by the angle *α* w.r.t. to the log G axis) defining a direction of projection together with a threshold value t∈R. Each log UV/G data point $x={\left(u,g\right)}^{T}\in R^{2}$ is then uniquely classified by fulfilling either $\langle x,s\rangle <t$ or $\langle x,s\rangle \geq t$, where 〈·,·〉 denotes the standard scalar product. We denote by $w_{\mathrm{UV}}$, $w_{G}$ and $w_{\mathrm{con}}$ the global linear separators that (by choosing appropriate values of *α* and *t*) separate the log UV/G data using the UV channel only, the green channel only and UV/G contrast (1:1), respectively. Furthermore, the separator $w_{F}$ uses the best UV/G contrast (not necessarily 1:1) by optimizing the Fisher criterion [36]. Note that for the Fisher criterion, contrary to local separation techniques where we tested for a set of discrete projection angles, arbitrary angles *α* are possible.

**Figure 8:**
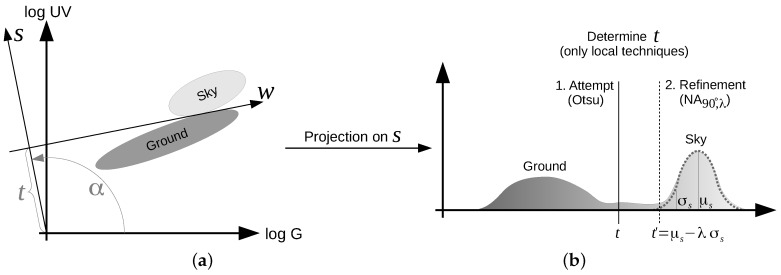
(**a**) (projection): Global and local separation techniques project the log UV/G data onto a hyperplane *s* with angle *α*. The angle *α* is always fixed for all global separation techniques, as well as the local technique ${\mathrm{NA}}_{\alpha ,\lambda }$. (**b**) (threshold selection): While the threshold value *t* is learned from a large set of training images for global separation techniques, local separation techniques learn it for each input image pair (log UV and log G) individually. Local separation techniques maximize Otsu’s criterion by iterating the threshold values *t*. Furthermore, the technique ${\mathrm{NA}}_{\alpha ,\lambda }$ approximates a normal distribution N(μ,σ) to the sky class (based on the threshold *t*) and refines the threshold value based on this distribution as $t^{\prime }=\mu -\lambda \sigma$, where *λ* is a correction factor.

The best threshold values *t* are chosen for all global linear separators based on the classification rates on all images of the recorded databases. In contrast, the thresholds of local linear separators need to be trained on each data input (the current UV and green images): For any data input, we first project the log UV/G data points on a hyperplane with angle *α* (as for the global separators). However, in contrast to global separation techniques, the threshold value *t* is learned individually for each data input by using Otsu’s method [37], an automated thresholding method that optimizes a criterion (in the following called the Otsu criterion) similar to the Fisher criterion. Afterwards, the input data are classified using the determined threshold value *t*. We found that the best results are achieved by using a more sophisticated method, in the following denoted by ${\mathrm{NA}}_{\alpha ,\lambda }$, which calculates a new threshold $t^{\prime }$ based on the first estimated threshold *t* as follows: After classifying the input data using the threshold value *t*, we fit a normal distribution N(μ,σ) to the data in the sky class. By setting a new threshold as $t^{\prime }=\mu -\lambda \sigma$, where *λ* is an appropriate correction factor, the classification rate can be increased. Note that the values of the projection angle *α* and the correction factor *λ* can be chosen arbitrary. We tested all combinations of values α∈{−45°,−40°,⋯,135°} and λ∈{0.1,0.2,⋯,5} for all datasets and chose the pair that maximized the classification rate. In our previous study [19], we tested a total of four different local separation techniques. Since they showed similar results on the newly-collected databases, we only show the results of ${\mathrm{NA}}_{90{}^{\circ},\lambda }$. The best results were achieved with a projection angle α=90° and λ∈[24] (depending on the database). However, the classification rate for λ∈[24] did influence the classification rates only minimally, such that λ=3 is a reasonable choice for arbitrary databases. A sketch of the linear separation technique ${\mathrm{NA}}_{90{}^{\circ},\lambda }$ can be found in Figure 8.

In our previous study [19], we showed that overfitting does not occur on the forest/suburban dataset for single channel separators by performing cross-correlation tests. This is to be expected, since the separator has only one degree of freedom (the threshold value) and the amount of collected data is huge. For contrast-based separators ($w_{F},w_{\mathrm{con}}$), there are up to two degrees of freedom (the threshold value and for the Fisher discriminant, the projection angle), such that the influence of training and test data is increased. Since we only collected a single day for each mineral skyline, we are not able to perform cross-correlation tests as previously done for the forest/suburban database; however, we believe that the total amount of data collected in this study now covers a wide range of ground objects and various sky conditions. Therefore, the learned values should generalize well to novel log UV/G data. Moreover, training is done on a randomly-drawn set of sample points from the respective database, while the tests are not only performed on a different set of sample points (Table 1), but also on the complete set of HDR images (UV and green, Figure 11).

Binary masks are non-linear separators that classify the log UV/G data by defining a classification for each possible combination of log UV/G data. Since we mapped the log UV/G data onto the discrete interval [0,255]∈N, a binary mask is a 256×256 B/W image where each color (black or white) specifies the classification of the corresponding log UV/G data. The mask for a dataset is based on the distribution of the log UV/G data (Section 2.2) and assigns to each possible combination of log UV/G data the class with the higher distribution. Note that the binary masks are the best possible non-linear separators if trained and tested on the same sample of log UV/G data. Therefore, the mask is overfitting the training data (the log UV/G data) on purpose to obtain an upper bound for the classification rate which can be obtained by a global (non-linear) separator.

## 3. Results

### 3.1. Records of Skylines

**Figure 9:**
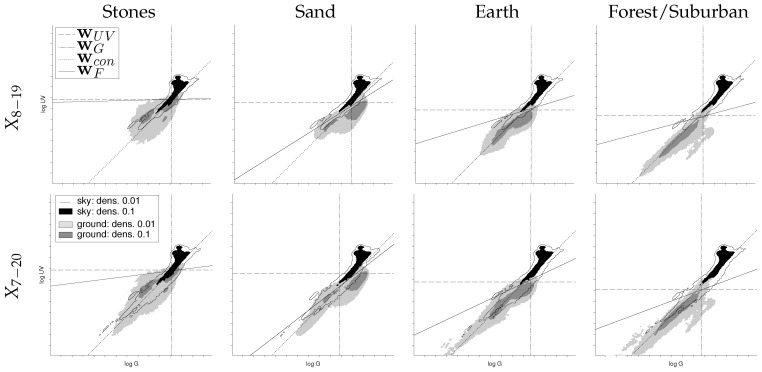
The columns show the log UV/G plots of the three mineral skylines (stones, sand, earth) and the skyline dominated by trees from the previous study (forest/suburban). Note that in each plot, the pooled sky data of all skyline databases are shown, representing a wide variety of weather conditions (the slightly different appearance is due to normalization). Each row shows the data recorded at day times between 8:00–19:00 and 7:00–20:00, respectively. The lines show the global linear separators that maximize the classification rate for each presented log UV/G dataset: $w_{\mathrm{UV}}$ (UV-only), $w_{G}$ (green-only), $w_{\mathrm{con}}$ (contrast: 1:1) and $w_{F}$ (contrast: Fisher discriminant). The classification rate of these separators can be found in Table 1. The black region and the black outline show the density level of the sky class for the levels 0.1 and 0.01 after transforming the point cloud into a continuous distribution by superimposing a Gaussian at each point (σ=1), respectively. Analogously, these levels are represented for the ground class by dark and light gray areas (Section 2.2). On each tick mark, the irradiance value is doubled, representing a ‘stop’ in camera terminology.

**Figure 10:**
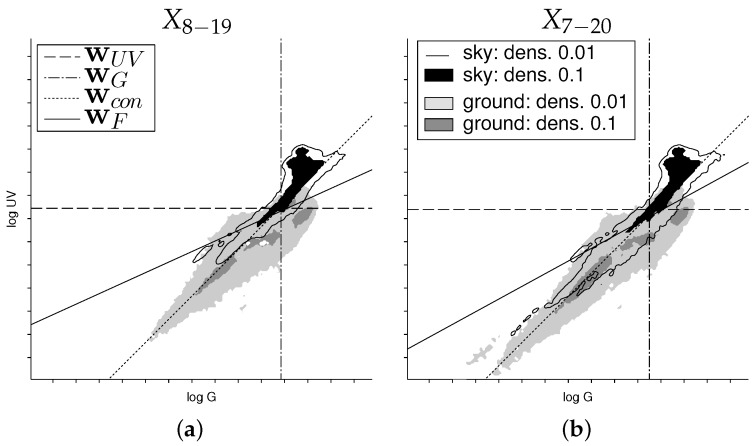
The plot shows the pooled data of all datasets recorded during the daytimes (**a**) 8:00–19:00 and (**b**) 7:00–20:00, where the same number of sample points has been taken from each of the four different datasets presented in Figure 9 (stones, sand, earth, forest/suburban). For details, see Figure 9.

The log UV/G plots of the three days recorded with different mineral skylines (stones, sand, earth), as well as the log UV/G plot of the forest/suburban dataset are shown in Figure 9. Note that only samples of the ground objects are restricted to the specific databases, while the sky data are always chosen from the complete sky data collected in all databases to enhance the variation of sky data as much as possible. A pooled log UV/G plot of all datasets can be found in Figure 10. As can be seen, all three mineral datasets show comparable distributions of the log UV/G data for objects; however, the UV portion in the UV/G contrast is higher in the stone skyline compared to sand and earth skylines. Furthermore, the ground objects form mainly two clusters (which can clearly be seen in Figure 13; compare Section 3.2). This is a consequence of the lighting conditions, since each log UV/G data point (mostly) corresponds either to an object lying in the direct sun (higher amount of green compared to UV) or in the shadows (higher amount of UV compared to green). On the recording days, the sky was mainly clear, such that purely diffuse lighting occurred only rarely.

Larger differences can be found by comparing the three mineral skylines to the data collected in the forest/suburban area. It can be seen that the overall brightness of the collected ground objects in the forest/suburban dataset is lower compared to the mineral skylines, since recordings also include multiple days with bad weather conditions. Furthermore, the forest/suburban data were collected over seven days with differing skylines with a wider variety of collected data, which leads to a higher spread in the corresponding log UV/G plot.

Except for the stone skyline, all plots show that the ground portion tends to have a higher amount of green light, while the sky portion has a higher amount of UV light. This supports the idea that a log UV/G contrast measure (not necessarily 1:1) can be used to classify the data.

#### 3.1.1. Global Separation Techniques

To evaluate the performance of each global separator on a specific database, we tested it on a test sample that differs from the training sample (even so, both are drawn from the same database). Table 1 shows that both the global linear separators $w_{\mathrm{UV}}$ (UV-only) and $w_{F}$ (Fisher discriminant) have classification rates close to the maximal achievable rates (binary masks, Section 2.3) for all datasets, except for the sand skyline dataset. However, in comparison to $w_{\mathrm{UV}}$ and $w_{F}$, the classification rates of $w_{G}$ and $w_{\mathrm{con}}$ are not competitive with values mainly in the range of 70%–85%. Besides the sand dataset, only a gain of 1% is achieved by using $w_{F}$ on log UV/G data instead of $w_{\mathrm{UV}}$ on the log UV data only. For the sand dataset, we found a high discrepancy between the classification rates achieved by $w_{\mathrm{UV}}$ and $w_{F}$ with 88.1% and 94.4% for dataset $X_{7–20}$ and 82.2% and 89.0% for dataset $X_{7–20}$, respectively.

**Figure 11:**
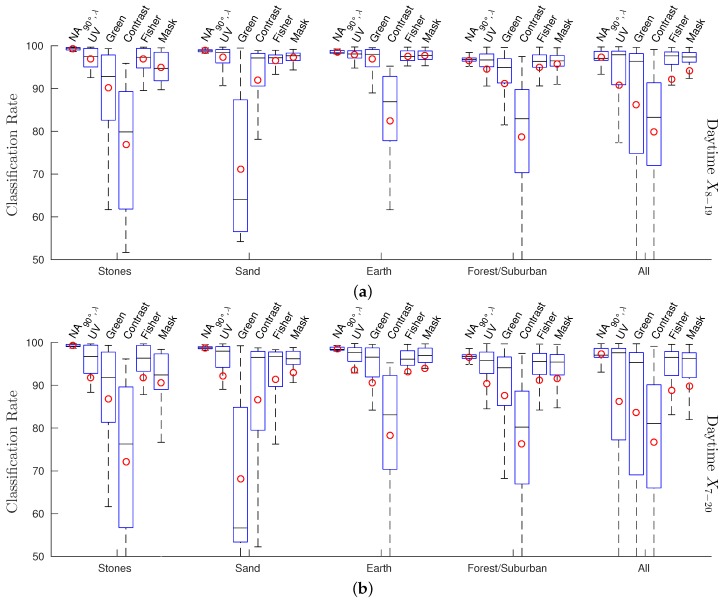
Box plots of the classification rates for the local separation technique ${\mathrm{NA}}_{90^{\circ },\alpha }$ and all global separation techniques (UV/green/contrast/Fisher/mask). As for the results presented in Table 1, the global separation techniques were trained on samples drawn from the specified databases, but in contrast, this evaluation uses single HDR image pairs (e.g., as captured by a mobile robot) as test data. The numbers of HDR image pairs classified in each box plot are for (**a**) $X_{8–19}$ and (**b**) $X_{7–20}$: stones, sand, earth: n=132/156; forest/suburban: n=924/1092; all: n=1320/1560. The box plots show the distribution of the classification rates for all separation techniques and databases. Each plot shows the mean (red circle), median (black line), the 25th and 75th percentiles (blue box) and a coverage of $3\sigma \hat{=}97.7\%$ (black dashed lines). For better readability, outliers are not shown.

For comparison with the local separation techniques, we used the separators learned for each dataset (using a sample of $10^{5}$ log UVG/G pixel pairs as the training set) and applied them to each captured HDR image pair (UV and green) directly. Figure 11 shows the mean, median, quartiles and the 3σ coverage of the classification rates of each image pair. The classification rates for the single HDR image pairs differ from the results shown in Table 1 for two reasons: First, the number of samples taken from the ground objects and sky are no longer equal, since the HDR image pairs contain an unequal amount of both classes. Second, the global separators were trained for a wide variety of weather and lighting conditions (for the sky data of all ten recorded days). This is not the case for single HDR image pairs, since each pair only contains the log UV/G data captured at one specific time. For example, an HDR image pair of the stone skyline during sunny weather can be classified more reliably, due to the higher contrast in the UV channel, than an HDR image pair of the forest/suburban database during rain. In both cases, the data contained in the HDR image pairs differ from the samples that contain the pooled data of the ground objects over several hours and the sky data of all ten recorded days. For databases collected on sunny days (e.g., the mineral skylines), this results in noticeably increased classification rates (e.g., 79% vs. 92% for global UV-only separation on the stone skyline). As can be seen, for the earth and forest/suburban databases, the median of the classification rates of the best global separation techniques (UV, Fisher, mask) is close to the median of the local separation technique (${\mathrm{NA}}_{90^{\circ },\alpha }$). However, for the day time 7:00–20:00 ($X_{7–20}$), the performance of global separators is noticeably worse compared to ${\mathrm{NA}}_{90^{\circ },\alpha }$.

We use bootstrapping [38] to test whether the mean classification rates of two separation techniques differ significantly (Table 2). The test set contains the classification rates of the two separation techniques for all HDR image pairs. Bootstrapping now generates a total of $10^{4}$ bootstrapping sets of the same size as the test set by redrawing from the test set (with repetition allowed). For each bootstrapping set, the difference between the mean classification rates for the two separation techniques is computed. Over all $10^{4}$ bootstrapping sets, we obtain a distribution of this difference. For this distribution, we test whether zero lies within the confidence interval (two-sided), using significance levels of 99.9%, 99% and 95%. If this is not the case, the difference of the mean classification rates is significant. The results show that for all separation techniques, the classification rates differ significantly, except for the global separators UV, Fisher and mask. It can be observed that for the mineral databases (stones, sand, earth), the global separation techniques UV and Fisher do not differ significantly, indicating that the performance of the techniques UV and Fisher are comparable.

**Figure 12:**
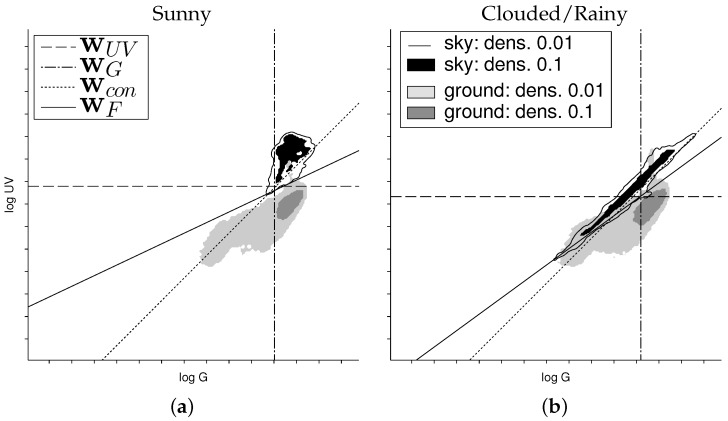
The plot shows the log UV/G data for the sand skyline together with the sky data (selected from all databases) of sunny days (**a**) and days where the sky was cloudy including rain (**b**). While the classification rate is similar in both cases using the Fisher discriminant $w_{F}$ (98.7% and 93.0%), it differs strongly for the UV-only separator $w_{\mathrm{UV}}$ (98.3% and 74.4%).

In Figure 12, we show two additional plots of the sand dataset, but using two different kinds of sky data: one time from sunny days and one time from cloudy and rainy days (selected from all skyline databases). While both classes can easily be separated using the sky data of the sunny days, achieving classification rates of 98.7% and 98.3% for $w_{\mathrm{UV}}$ and $w_{F}$, respectively, the classification gets more demanding when using the sky data of the cloudy/rainy days. In this case, $w_{\mathrm{UV}}$ has a classification rate of 74.4%, while $w_{F}$ still achieves 93.0%. We could observe this big difference between $w_{\mathrm{UV}}$ and $w_{F}$ only in this special setup (sand skyline under cloudy/rainy weather conditions for the sky portion), in all other combinations they showed comparable performances.

#### 3.1.2. Local Separation Techniques

To verify the results of our previous study [19], we used the local separation techniques from Section 2.3 to classify the log UV/G data for each single image (unsupervised learning). The results are presented in Figure 11 and show a superior performance to the global separation techniques by achieving a classification rate of approximately 95%–99% on all databases. As described, for the local separation technique ${\mathrm{NA}}_{\alpha ,\lambda }$, the separation angle is fixed, but to determine the optimal angle *α* (for a specific dataset), we calculated the classification rates for fixed values of *α* and chose the value maximizing the classification rate. We found that the best performances are achieved for $\alpha \approx 90^{\circ }$ (Section 2.3). The correction values *λ* used by ${\mathrm{NA}}_{90^{\circ },\alpha }$ showed the best performances were between 3.0 and 3.6 for the mineral skylines and 2.0 for the forest/suburban skyline. While the correction value *λ* should be chosen depending on the environment and weather conditions to achieve the best results, it still outperformed all other methods for all values λ∈[2,4] on the tested databases.

### 3.2. Records of Ground Objects

**Figure 13:**
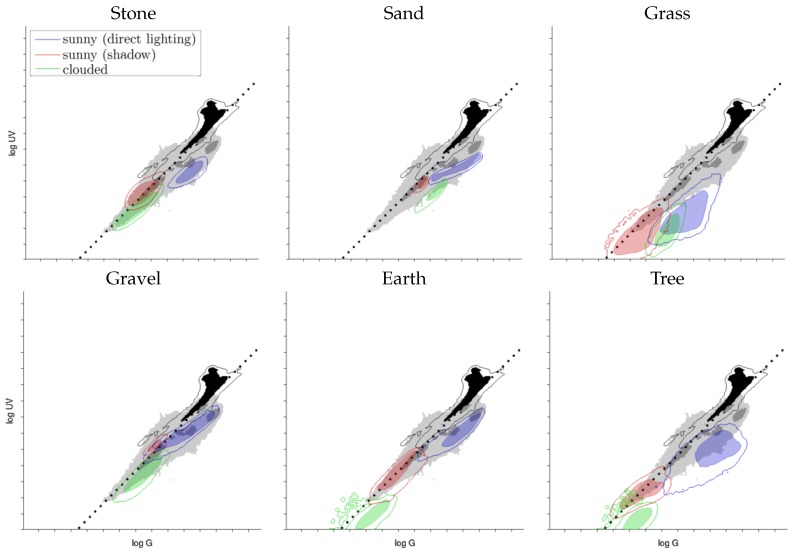
A total of 61 different samples of ground objects has been collected. This plot shows six different ground objects, each recorded under three different conditions: the object lies in the sun (blue) or in the shadow (red), both on a sunny day, or the object was recorded on a cloudy day (green). For comparison, the pooled data (ground and sky) from Figure 10 ($X_{7–20}$) are shown together with the best 1:1 contrast separator ($w_{\mathrm{con}}$, dotted line). Examples of the log UV/G data used to create the sand and grass plots are presented in Figure 6.

**Figure 14:**
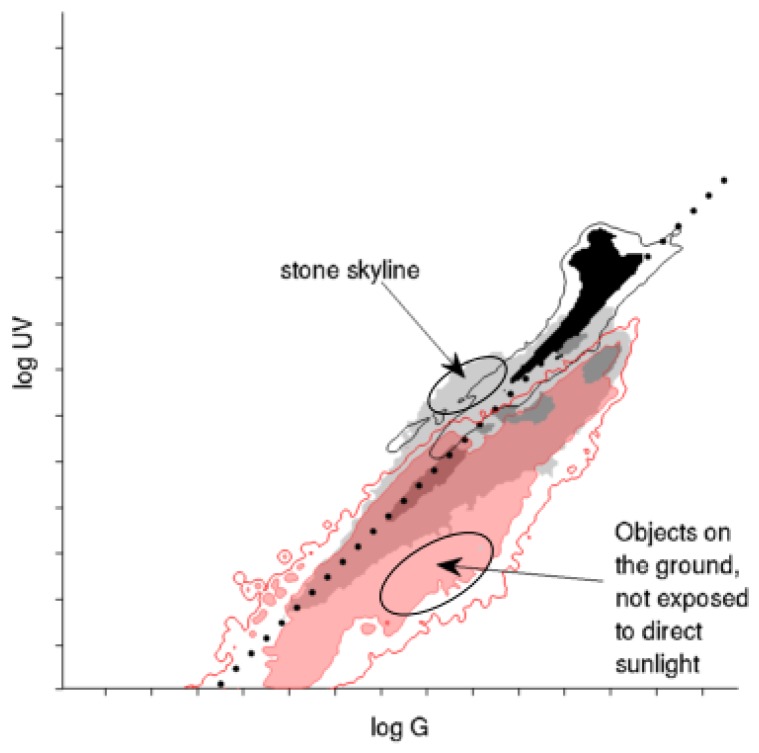
Pooled plot of all ground object databases (red). The wide variety of different objects and lighting conditions (partly in the same image) does not allow a unique distinction of the lighting conditions as in Figure 13. For comparison, the pooled data from Figure 10 ($X_{8–19}$) are shown. The highlighted areas (ellipses) show where differences between the object and skyline databases occur.

To get a visual impression of single ground objects (without sky) under different lighting conditions, compared to the datasets of the skylines collected over the complete days under bright and dry weather conditions, we collected a total of 61 different single samples of ground objects and visualized them together with the data collected over the complete days. The results are presented in Figure 13 and Figure 14.

As can clearly be seen from the log UV/G plots, the different ground objects differ strongly regarding overall brightness, UV/G contrast and spread of those values. A strong influence of the overall lighting condition is visible in the data. First, due to the indirect lighting by the blue sky and the high reflectivity for even small wavelengths (UV/near UV; see [39]), the log UV/G contrast of stones and gravel in shadow is high (a higher amount of UV light compared to green light). In comparison, grass and trees reflect green light around three-times better than UV light [40], such that the log UV/G contrast is lower. Interestingly, sand was the only mineral that also reflected UV light at a lower rate compared to stones, gravel and earth. Second, ground objects under direct sunlight show a higher proportion of green light compared to UV for all recorded materials. Except for gravel and earth, which show a small overlap with the 1:1 contrast in Figure 13, all other materials are clearly separable from the sky using a linear contrast separator. Third, on cloudy days, where objects were mainly illuminated by diffuse light reflected from the cloud-covered sky, the collected data show a similar behavior like objects under direct sunlight on sunny days: the data for all materials show a dominance of green light (probably due to a reduction of UV irradiation by the cloud cover); however, the overall less strongly lit scenes lead to a strong decrease of the irradiance values on both axes. In summary, diffuse illumination of mineral objects by the blue sky is the most challenging condition for global separators.

Figure 14 shows a log UV/G plot of the object database. The wide variety of different objects and lighting conditions (partly in the same image) does not allow a unique distinction of the lighting conditions as in Figure 13; therefore, the data of the complete object database are pooled. The log UV/G plot might give the impression that the collected objects have lower UV values compared to the skyline databases. This is due to the overall smaller amount of direct sunlight in most scenes where the object database has been recorded (forest, bushes, etc.) compared to the skyline databases, which were mostly recorded in well-lit scenes.

### 3.3. Panoramic Images

**Figure 15:**
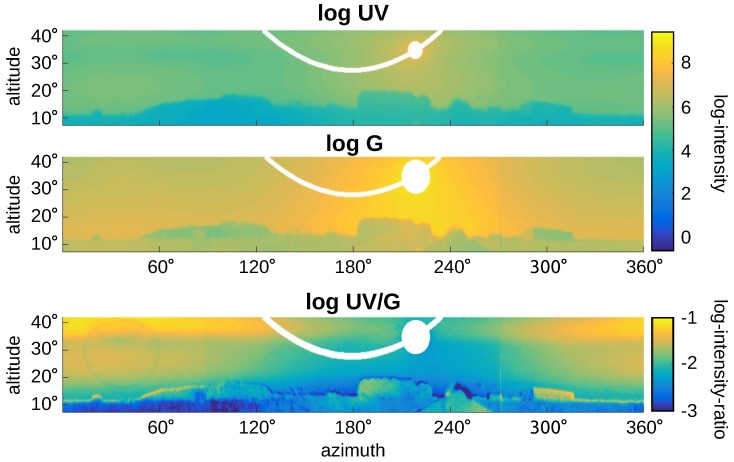
A panoramic image with clear blue sky in the background has been captured at 17:25 on a sunny day in the vicinity of Bielefeld University on 12 May 2016. The upper two images show the log UV and log G intensities, respectively. The bottom plot shows the log UV/G ratio between both channels. White areas are corrupted by direct sunlight shining onto the sensor and are masked out.

**Figure 16:**
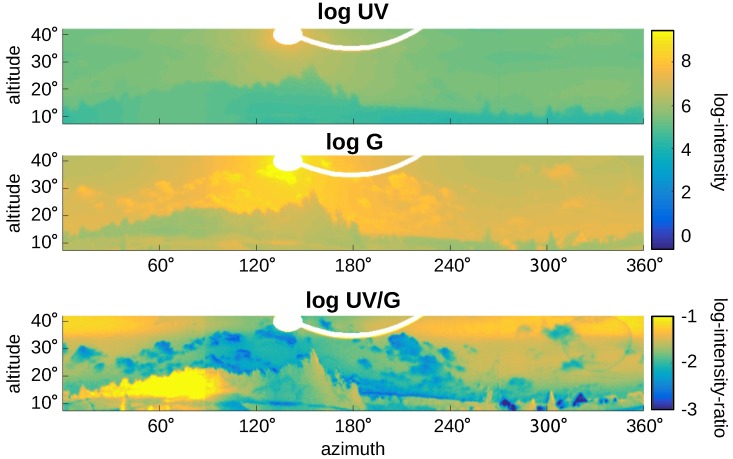
A panoramic image of fields and forest in front of a clouded sky has been captured at 18:30 on a sunny day in the vicinity of Bielefeld University on 27 June 2016 (for additional information, compare Figure 15). Note that while there are many clouds in the sky, the Sun itself is not covered by clouds. The performance of the best global and local separation techniques tested in this study are shown in Figure 17.

Figure 15 and Figure 16 show panoramic images captured on sunny days in the vicinity of Bielefeld University. They show the raw (non-normalized) log UV and log G values obtained by the HDR algorithm and their ratio $\log\frac{\mathrm{UV}}{G}=\log\mathrm{UV}-\log G$, denoted by log UV/G. The blue sky in Figure 15 shows a gradient of UV and green light over the sky [41,42], while Figure 16 shows the strong influence of clouds. Unfortunately for global separation techniques, also the log UV/G contrast differs strongly between data points close to the sun compared to data points opposite to the sun, increasing the difficulty of applying a global threshold. The experimental setup used to capture the skyline databases was always pointing in the same direction (mostly north); however, due to the movement of the Sun, the appearance of the sky differs strongly over the day. The same effect appears for local separation techniques; however, they can adapt their thresholds for smaller segments of the image individually, which is not possible for global separation techniques.

**Figure 17:**
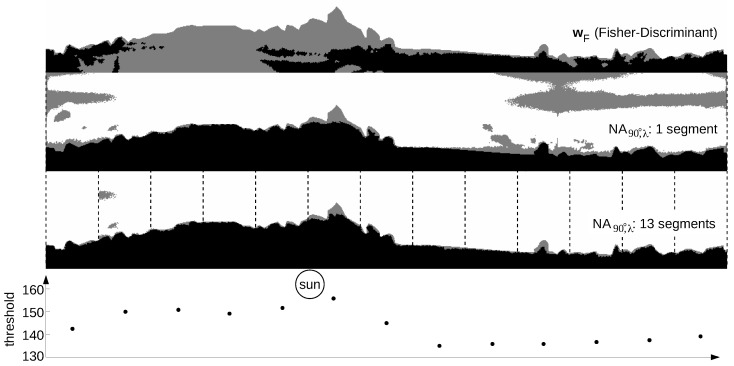
Example of a skyline extraction from the panoramic log UV/G image shown in Figure 16 using the separation techniques $w_{F}$ (global) and ${\mathrm{NA}}_{90^{\circ },\alpha }$ (local, UV-only) (middle, bottom). Pixels classified as sky and ground are colored white and black, respectively, while gray pixels indicate misclassifications. The gradient of the log UV and log G data over the sky shown in Figure 15 and Figure 16 increases the difficulty for local separation techniques to find a threshold value to separate both classes on the whole image. By splitting the panoramic image into smaller images (bottom; here $28^{\circ }$ steps), this problem can be avoided. The graph shows the threshold values for each individual segment. Contrary to local separation techniques, global separation techniques cannot be individually adapted for single segments. The classification rates from top to bottom are 82.1%, 87.4% and 98.0%.

Figure 17 shows how the global separation technique $w_{F}$ and the local separation technique ${\mathrm{NA}}_{90^{\circ },\alpha }$ (UV-only separation) compare on the panoramic image with direction-dependent lighting conditions shown in Figure 16. As can be seen, the global separation technique (classification rate: 82.1%) misclassifies around regions that are brightly lit by direct sunlight, while the local separation technique (classification rate: 87.4%) misclassifies large regions of the sky that are opposite of the Sun. For the latter, this problem can be avoided by using different thresholds for different parts of the panoramic image. Here, we calculated the threshold values for a total of 13 segments (each spanning an azimuthal angle of around $28^{\circ }$), which increases the classification rate to 98.0% (for this image). The number of segments has been chosen as a trade-off between too few segments (no adaptation to differing lighting conditions) and too many segments (small segment sizes might lead to unstable results from Otsu’s method). The skyline extraction could furthermore be smoothed by linearizing the threshold values between neighboring segments, resulting in an individual threshold for each column.

The classification rates for in total 12 recorded panoramic images can be found in Table 3. We divided them into two groups; one group where direct sunlight is shining onto the sensor (e.g., Figure 15 and Figure 16) and one where the Sun is covered by clouds. As can be seen, the classification rates are stable for the tested separation techniques as long as the Sun is covered by clouds. However, if direct sunlight is shining onto the sensor, the classification rates drop for the global separation technique $w_{F}$ from 92% down to 83% and for the local separation technique ${\mathrm{NA}}_{90^{\circ },\alpha }$ (without slicing the panoramic image into multiple segments), from 94% down to 87%. In contrast, by calculating an individual threshold for multiple segments (here: 13), the classification rate of ${\mathrm{NA}}_{90^{\circ },\alpha }$ stays stable at around 97%.

## 4. Discussion

### 4.1. Skyline Separation Using Global Separation Techniques

As expected, the results show that the newly-collected mineral skyline databases have a higher portion in the UV range compared to the forest/suburban skyline database. In brightly-lit scenes (e.g., afternoons on sunny days), the log UV/G values between minerals (except for the stone skyline) and sky differ noticeably and allow a coarse classification by a linear separator (Figure 9) as for the forest/suburban database. However, for indirectly-lighted minerals (objects in shadow, clouds in front of the Sun), the classification becomes more difficult. Due to the higher UV reflectivity, ground objects in the mineral skyline databases are generally closer to the sky data. Furthermore, the increased UV intensity (compared to the green light) caused by the indirect lighting of the sky leads to a high amount of data points that have a log UV/G contrast where the UV portion dominates. Especially for the stone skyline, a linear 1:1 contrast separator cannot be used anymore. This result is contrary to the hypothesis that a global linear separation can best be achieved by using a log UV/G contrast.

Interestingly, we found that under specific circumstances, the quality of classification for the sand skyline can be increased noticeably by using log UV/G contrast: As can be seen in Table 1, the classification rates are increased by ≈6% compared to UV-only separation and even more by ≈30% compared to G-only separation. The achieved classification rates were only ≈2% worse compared to the binary masks, which represent the best global separation. However, the increased classification rates depend strongly on the weather conditions. As shown in Figure 12, the advantage of using log UV/G data is only prominent if the (dry) sand skyline is visible in front of a cloudy/rainy sky. If instead the sky of a sunny day is used, the UV-only and UV/G contrast separations achieve about the same classification rates.

The data collected in the skyline databases cover four specific environments (stones, sand, earth, forest/suburban); however, an insect, e.g., an ant navigating through undergrowth, might encounter a wider variety of ground objects. The latter should be covered well by the object databases. To compare the collected log UV/G data from the skyline and object databases, Figure 14 shows a pooled plot of the object databases compared to a pooled plot of the skyline databases. As can be seen, the log UV/G data of ground objects from the object and the skyline databases intersect mostly, except for two areas (Section 3.2): Under strong illumination, the ground class of the stone skyline database partially extends into the sky class. The ground class of the object database extends into lower log UV/G values (in some cases, less strong illumination conditions during the recording, e.g., in forests). However, neither do the log UV/G values of the ground database reach or exceed those of the objects from the stone skyline, nor do we observe an increased overlap of the ground database with the sky class of the skyline databases. Therefore, it can be assumed that global separation techniques will exhibit the same performance on the ground database as on the collected skyline databases.

The position of the Sun strongly influences the appearance of the scene, especially of the sky class. An additional adaptation of the global separators to the position of the Sun relative to the camera heading would be necessary to provide a reliable separation between the sky and ground class. However, this would undermine the idea of a fixed global threshold as a simple separation technique. In contrast, local separation techniques can easily be adapted to different illuminations by splitting the image into segments and finding individual thresholds, making them a preferred choice for scenes with highly direction-dependent lighting conditions.

We could only observe one special and presumably unlikely situation, sun-lit sand in front of dark clouds (Figure 12), in which a global separation technique noticeably benefits from a log UV/G contrast mechanism compared to UV-only separation. Therefore, the benefits of dual channel separation are too small that we believe that insects would not profit by a global log UV/G separator. Considering day times without dawn or dusk only ($X_{8–19}$), it can be seen that the global separation technique $w_{F}$ achieves classification rates comparable to the local separation method. However, considering day times including dawn and dusk ($X_{7–20}$), it can be seen that local separation methods outperform all global separation methods. Therefore, global separation methods do not seem to be reasonable choices for skyline extraction in robot applications; instead, local separation methods should be used.

### 4.2. Skyline Separation Using Local Separation Techniques

As discussed in Section 4.1, we have found an increased UV portion for stones, sand and earth objects in the mineral skyline databases compared to the suburban/forest skyline database. For global separation techniques, the increased UV portion lessens the quality of classification between ground objects and the sky. However, for local separation techniques, this effect could not be observed; the classification rates of the local separation techniques are around 96%–99% for all databases and tested techniques. This confirms the idea proposed by Wehner [16] (p. 96) that a classification between skyline and ground can be obtained by using the UV channel only and that a local (adaptive) threshold performs best.

### 4.3. Omnidirectional Skyline Extraction

As shown by [20], local UV-only separation can be used to extract the skyline from panoramic images in real time in robot applications using similar computationally-inexpensive methods (e.g., watershed algorithm). They demonstrate that an agent can use this segmented skyline information to localize itself on a previously-driven track. In [6], this idea was enhanced by adding rotational invariance, such that even aggressively-maneuvering robots are able to localize themselves on a previously-driven track. Using the adaption to omnidirectional images by using multiple segments, it may be possible to improve the performance in such applications. Finally, the findings of this study indicate that the results of [6,20] will be valid for a wide range of environments, making skyline extraction using UV-only imaging a valuable tool for robot applications.

## 5. Conclusions

The results of our previous work [19] showed that for global separation techniques, the classification could only be slightly improved using two different color channels, namely UV and green. Additionally, the test of local separation techniques showed that using both UV and green color channels does not improve the classification quality over UV-only classification. Due to the limited range of data collected in the previous study (only suburban and forest skylines), the variety of collected data is increased by adding mineral skylines (stones, sand, earth), as well as a collection of several ground objects (shrubs, fir sprigs, straw) in this study.

We could show that the mineral skylines have different log UV/G irradiance characteristics, which increases the difficulty of a global classification based on the log UV/G data. The stone skyline database showed a high irradiance in the UV range, such that a distinction from the sky is generally difficult. The same holds for the earth skyline database; however, the irradiance in the UV range was less strong compared to the stones. Only for the special case of dry sand in front of a cloudy and/or rainy sky, the classification rate for the global linear separators could be improved noticeably by using log UV/G data instead of log UV data only. In all other cases, the use of log UV/G data did improve the classification rates only slightly.

For the local separation techniques, we could verify the results from the previous study: Independent of the type of skyline (minerals or plants), the best classification rates could be achieved by using the log UV data only. Moreover, we could show that local separation techniques can be easily adapted to work with panoramic images by splitting them into multiple segments. This makes local separation techniques on UV images a promising tool for skyline extraction in autonomous navigation for a wide range of environments.

## Figures and Tables

**Table 1:** The classification rates of the global separators introduced in Section 2.3—the linear separators $w_{\mathrm{UV}}$ (UV-only), $w_{G}$ (green-only), $w_{\mathrm{con}}$ (contrast: 1:1), $w_{F}$ (contrast: Fisher discriminant) and the binary masks (non-linear UV/G separator)—applied to the datasets shown in Figure 9 and Figure 10. The separation methods were trained and tested on two different samples (each containing $10^{5}$ elements of the sky and ground class) drawn from the specified databases. An evaluation using single HDR image pairs (e.g., as captured by a mobile robot) as test data instead can be found in Figure 11. Only for sand (highlighted), the classification rates show a noticeably increased performance for the UV/G contrast (Fisher discriminant) compared to UV-only separation. In all other cases, both methods show a similar performance, both slightly worse compared to the best possible performance of the binary masks. Classification rates obtained by applying the learned global separation techniques to the input HDR images (UV and green) directly can be found in Figure 11.

**Global**	$X_{8–19}$				
	**UV**	**Green**	**Contrast**	**Fisher**	**Mask**
Stones	84%	78%	70%	84%	88%
**Sand**	88%	60%	89%	94%	95%
Earth	94%	88%	78%	94%	95%
Forest/Suburban	95%	92%	80%	96%	97%
All	88%	79%	79%	89%	92%
**Global**	$X_{7–20}$				
	**UV**	**Green**	**Contrast**	**Fisher**	**Mask**
Stones	79%	75%	67%	79%	84%
**Sand**	83%	60%	86%	89%	91%
Earth	89%	84%	73%	88%	90%
Forest/Suburban	91%	89%	77%	92%	93%
All	83%	75%	75%	84%	87%

**Table 2:** Significance tests were performed on all skyline datasets between 7:00 and 20:00 to compare their classification rates. We used bootstrapping [38] as the statistical test; for details, see Section 3.1.1. The shown significance values are 99.9% (***), 99% (**) and 95% (*); for the databases forest/suburban and all, all significance tests are highly significant (99.9%).

**Stones**	${\mathrm{NA}}_{90^{\circ },\lambda }$	**UV**	**Green**	**Contrast**	**Fisher**	**Mask**
${\mathrm{NA}}_{90^{\circ },\lambda }$		***	***	***	***	***
UV			***	***	-	-
Green				***	***	***
Contrast					***	***
Fisher						-
Mask						
**Sand**	${\mathrm{NA}}_{90^{\circ },\lambda }$	**UV**	**Green**	**Contrast**	**Fisher**	**Mask**
${\mathrm{NA}}_{90^{\circ },\lambda }$		***	***	***	***	***
UV			***	***	-	***
Green				***	***	***
Contrast					***	***
Fisher						-
Mask						
**Earth**	${\mathrm{NA}}_{90^{\circ },\lambda }$	**UV**	**Green**	**Contrast**	**Fisher**	**Mask**
${\mathrm{NA}}_{90^{\circ },\lambda }$		***	***	***	***	***
UV			***	***	-	**
Green				***	***	***
Contrast					***	***
Fisher						-
Mask						

**Table 3:** We recorded a total of 12 panoramic images and divided them into two groups; one group where direct sunlight is shining onto the sensor (e.g., Figure 15 and Figure 16) and one where the Sun is covered by clouds. The classification rates for the separation techniques tested on each panoramic image, as well as the average classification rate for each group is shown; the column representing the results for the skyline shown in Figure 17 is highlighted (bold).

Separation-Technique	Sunlight Conditions												Average	
	Covered (C)						Direct (D)						C	D
$w_{F}$	99%	97%	98%	97%	95%	67%	97%	**82%**	81%	79%	78%	82%	92%	83%
${\mathrm{NA}}_{90^{\circ },\alpha }$, 1 Seg.	99%	91%	98%	98%	94%	82%	99%	**87%**	72%	91%	85%	87%	94%	87%
${\mathrm{NA}}_{90^{\circ },\alpha }$, 13 Seg.	99%	97%	97%	97%	98%	92%	98%	**98%**	99%	96%	97%	97%	97%	97%
